# Feasibility and impact of an evidence-based electronic decision support system for diabetes care in family medicine: protocol for a cluster randomized controlled trial

**DOI:** 10.1186/1748-5908-8-83

**Published:** 2013-08-05

**Authors:** Annemie Heselmans, Stijn Van de Velde, Dirk Ramaekers, Robert Vander Stichele, Bert Aertgeerts

**Affiliations:** 1 Academic Centre for General Practice, Department of Public Health and Primary Care, Katholieke Universiteit Leuven, Kapucijnenvoer 35 blok d, Leuven 3000, Belgium; 2 Department of Public Health and Primary Care, Kapucijnenvoer 35 blok d, Leuven 3000, Belgium; 3 Belgian Center for Evidence-Based Medicine, Belgian Branch of the Dutch Cochrane Collaboration, Kapucijnenvoer 33 blok j, Leuven 3000, Belgium; 4 EBMPracticeNet, Kapucijnenvoer 33 blok j, Leuven 3000, Belgium; 5 ZNA Hospital Network Antwerp, Leopoldstraat 26, Antwerp 2000, Belgium; 6 Department of Pharmacology, Universiteit Gent, De Pintelaan 185, Ghent 9000, Belgium

**Keywords:** Diabetes mellitus, Decision support systems, Clinical, Point-of-care systems, Electronic health records, Guideline implementation

## Abstract

**Background:**

In Belgium, the construction of the national electronic point-of-care information service, EBMPracticeNet, was initiated in 2011 to optimize quality of care by promoting evidence-based decision-making. The collaboration of the government, healthcare providers, Evidence-Based Medicine (EBM) partners, and vendors of Electronic Health Records (EHR) is unique to this project. All Belgian healthcare professionals get free access to an up-to-date database of validated Belgian and nearly 1,000 international guidelines, incorporated in a portal that also provides EBM information from sources other than guidelines, including computerized clinical decision support that is integrated in the EHRs.

The EBMeDS system is the electronic evidence-based decision support system of EBMPracticeNet. The EBMeDS system covers all clinical areas of diseases and could play a crucial role in response to the emerging challenge posed by chronic conditions. Diabetes was chosen as the analysis topic of interest. The objective of this study is to assess the effectiveness of EBMeDS use in improving diabetes care. This objective will be enhanced by a formal process evaluation to provide crucial information on the feasibility of using the system in daily Belgian family medicine.

**Methods:**

The study is a cluster-randomized trial with before/after measurements conducted in Belgian family medicine. Physicians’ practices will be randomly assigned to the intervention or control group in a 1:1 ratio, to receive either the EBMeDS reminders or to follow the usual care process. Randomization will be performed by a statistical consultant with an electronic random list generator, anonymously for the researchers. The follow-up period of the study will be 12 months with interim analysis points at 3, 6 and 9 months. Primary outcome is the one-year pre- to post-implementation change in HbA1c. Patients will not be informed about the intervention. Data analysts will be kept blinded to the allocation.

**Discussion:**

The knowledge obtained in this study will be useful for further integration in other Belgian software packages. Users’ perceptions and process evaluation will provide information for improving the feasibility of the system.

**Trial registration:**

The trial is registered with the ClinicalTrials.gov registry: NCT01830569.

## Background and rationale

Evidence is of no use if it remains buried in the literature and is not implemented in practice. A first step is the conversion of evidence from studies into clinical practice guidelines. Point-of-care decision support systems based on electronic guidelines have been suggested to successfully deliver the knowledge embedded in Evidence-Based (E-B) guidelines [1,2]. A number of studies have already shown positive findings for some types of decision support systems such as drug-dosing systems and computer-based reminder systems for preventive care services [3,4]. The evidence is less clear for more complex electronic guideline implementation systems [5-9].

In Belgium, the construction of the national electronic point-of-care information service EBMPracticeNet was initiated in 2011 to optimize quality of care by promoting evidence-based decision-making. The National Institute for Health and Disability Insurance (INAMI-RIZIV) provides funding for this organization. The collaboration of the government, healthcare providers, Evidence-Based Medicine (EBM) partners, and vendors of Electronic Health Records (EHR) is unique to this project. All Belgian healthcare professionals get free access to an up-to-date database of validated Belgian and nearly 1,000 international guidelines, incorporated in a portal that also provides EBM information from sources other than guidelines, including computerized clinical decision support that is integrated in the EHRs. The link between all the EHRs from different vendors and a national database held on a single platform and controlled by all EBM organizations in Belgium is the strength of EBMPracticeNet [10].

The EBMeDS system is the electronic evidence-based decision support system of EBMPracticeNet. The EBMeDS system was developed by Duodecim [11] and will gradually be integrated in the different Belgian software packages. EBMeDS is currently being integrated in the software of HealthOne, one of the Belgian electronic medical record systems (EMR).

The main distinguishing feature of the EBMeDS system is its intuitive approach and its platform-independent, service-oriented architecture, with strong central management. This has been recognized as being critically important for keeping the evidence up-to-date and for integrating the system in the different software packages used by all physicians involved in the care processes. In addition, the involvement of end-users during the implementation process is a critical feature of its success [12,13].

The EBMeDS system covers all clinical areas of diseases and could play a crucial role in response to the emerging challenge posed by chronic conditions such as hypertension, diabetes, and heart failure, etc. Chronic conditions increasingly affect the population and could lead to serious complications and disability in the long term if not managed appropriately in a proactive healthcare system. Chronic conditions require collaboration between different healthcare workers, recurrent visits, adherence to (sometimes overlapping or diverging) care plans and long-term disease and treatment monitoring [5]. Theoretically, the EBMeDS system has the potential to deal with these multifactorial dimensions of chronic diseases in ambulatory settings.

As with any health intervention, rigorous testing is warranted to determine whether the EBMeDS system could realize improvements in chronic care processes and, more especially, patient outcomes. Based on the results of a previous qualitative study in a pilot setting, we adapted the mode of evidence delivery and the quantity of reminders of a previous version of the system to prevent reminder fatigue [14].

Diabetes was chosen as the analysis topic of interest. Type II diabetes mellitus is a highly prevalent disease and at the moment the leading cause of blindness, renal dysfunction, non-traumatic limb amputation, and decreased life expectancy [15]. Optimal care of diabetes patients has been difficult to achieve because of the difficulties in sustaining regular monitoring and attention to many risk factors over many years [16]. Clear recommendations on the basis of high levels of evidence exist to prevent these complications, but there is still a large gap between recommended care and the care that patients actually receive [17,18].

The objective of this study is to assess the effectiveness of EBMeDS use in improving diabetes care as a case study. This objective will be enhanced by a formal process evaluation to provide crucial information on the feasibility of using the system in daily Belgian family medicine.

## Methods

The study is a cluster-randomized trial with before/after measurements conducted in Belgian family medicine. A cluster design is necessary to minimize contamination that might occur if individual patients were randomized. Randomization will be performed at practice level; the intervention will happen at physician level; and data will be analyzed at patient level, in a multilevel analytical approach. The follow-up period of the study will be 12 months.

Physicians’ practices will be randomly assigned to the intervention or control group in a 1:1 ratio, to receive either the EBMeDS reminders or to follow the usual care process.

### Participants and setting

Physicians eligible to participate in the study are all family physicians (739 Dutch-speaking and 891 French-speaking) using HealthOne (1,630 family physicians). Physicians will be included if the following criteria are met:

1. The physician has integrated HealthOne in his consultations;

2. The physician uses the functionality of e-prescribing within HealthOne so that prescribed medication can be identified from the electronic medical records; and

3. The physician agrees to be involved in the study.

Patients are included if:

1. They are 18 years or older.

2. They have their electronic medical records registered with one of the participating family physicians. Medical records are not centralized in Belgium. Patients are free to choose any physician, and can even see several physicians at the same time. To encourage a patient to have their medical records held by a single physician, a voluntary enlisting program was introduced (Global Medical File) a decade ago. For every enlisted patient, the general practitioner who keeps their central medical records receives a registration fee. Enlisted patients are reimbursed to a larger extent by the health insurance fund.

3. They are enlisted and have an established diagnosis of diabetes at the baseline time point of the study (identified as having: an ICPC code of diabetes; or a prescription for a diabetes-specific drug; or the necessary laboratory results to confirm diabetes).

Exclusion criteria are pregnancy and cognitive impairment.

### Interventions

#### Trial preparation

To ensure that the point-of-care information provided through the EBMeDS system is context-sensitive, EBMPracticeNet had the responsibility to tailor the diabetes reminders and suggestions. Diabetes reminders were adapted to the Belgian context in collaboration with Domus Medica, a Belgian organization delivering evidence-based information for primary care. This adaptation process also included careful selection of the most relevant messages for the Belgian family physician, taking into account the risk of alert fatigue.

### The EBMeDS system in the intervention groups

The EBMeDS system receives structured patient data from the electronic medical records in HealthOne and returns reminders, therapeutic suggestions, and diagnosis-specific links to guidelines. Electronic forms and calculators (*e*.*g*., a calculator for glomerular filtration) are integrated in the system.

The original EBMeDS system covers a full spectrum of all clinical areas. Relevant reminders in all clinical areas are shown to the physicians in the intervention group, but analysis is limited to the treatment and follow-up of diabetes patients.

HealthOne is one of the electronic medical record systems in Belgium. The software enables physicians to record patient histories and contacts, to display test results, to access generated notes and reports, and to support physicians in decision-making for patient care.

### The evidence Linker in the control and intervention groups

The Evidence Linker has been integrated in Belgian routine practice since 2012 and could be considered part of the usual care process. When entering a diagnosis coded in ICPC, relevant clinical practice guidelines are retrieved by the Evidence Linker and can by consulted on the initiative of the family physician.

The Evidence Linker service is offered by the Belgian Centre for Evidence-Based Medicine (CEBAM) and is available to all physicians in the control group as well as in the intervention group during study follow-up.

The content and recommendations of the diabetes guidelines presented in the Evidence Linker are similar to the content of the EBMeDS diabetes reminders. Differences between the intervention and control groups are the method of evidence delivery (‘push’ versus ‘pull’) and the format of the recommendations (‘guideline format, long’ versus ‘reminder format, short’).

### Objectives

The objective of this study is to assess the feasibility of the use of the EBMeDS system in daily Belgian family medicine and to study the effectiveness of EBMeDS use in improving diabetes care according to current evidence-based guidelines. Specific research questions are:

1. Do family physicians use the EBMeDS system in daily practice?

2. Does the use of the EBMeDS system by family physicians lead to an improvement in diabetes control compared to the control group?

3. How do family physicians perceive the reminders included in the EBMeDS system?

### Outcomes

#### Baseline measurements

Baseline demographic data on patient age, gender, years since the diagnosis of diabetes, nursing home residence (yes/no), HbA1c, cholesterol levels, and blood pressure will be collected. Data on physician age, gender, years of experience, and native language will be requested as well.

### Primary and secondary outcomes

Primary outcome is the one-year pre- to post-implementation change in HbA1c.

Secondary outcomes are the one-year differences in cholesterol levels and systolic and diastolic blood pressure. A composite patient score and a composite process score are calculated representing the change in diabetes (process) control and associated cardiovascular risk [19]. The composite patient score consists of reaching the evidence-based targets for glycated hemoglobin, blood pressure (systolic and diastolic), and cholesterol. The process composite score consists of meeting the evidence-based targets of the number of blood pressure measurements, the number of laboratory results of HbA1c, cholesterol and micro-albuminuria, a prescription of statin (yes/no), if high cardiovascular risk a prescription of aspirin/clopidogrel (yes/no), if hypertension or nephropathy ACE inhibition/sartan (yes/no).

Outcomes that cannot be reliably detected from the electronic medical records such as smoking status, and feet and eye examinations are excluded from the composite scores, as well as outcomes in which family physicians’ influences are minimal (*e*.*g*., BMI, physical activity, etc.).

Outcomes of the composite scores with their respective targets are shown in Table 1. Recommendations and targets are based on the diabetes guidelines of Domus Medica [20] and the Belgian ‘Zorgtraject diabetes’ [21].

**Table 1:** Variables included in the composite scores

***Variable***	***Process target***	Maximum score per period*	***Clinical target***	***Maximum score per period***^**†**^
HbA1c	Quarterly	2	< 7%	1
Blood pressure	Quarterly	2	< 130/80 mm Hg	1
Cholesterol (LDL, HDL, Total)	Annual	1	LDL < 100 mg/dL	1
Microalbuminuria check	Annual	1		
A prescription of statin	Yes/no	1		
If high cardiovascular risk, a prescription of aspirin/clopidogrel	Yes/no	1		
If hypertension or nephropathy, ACE inhibition/sartan	Yes/no	1		
Total		9		3

*Maximum score for process composite per period = 9 (7 variables).

^†^ Maximum score for patient composite per period = 3 (3 variables).

Each of the parameters in the composite scores is compared with their respective target. Each outcome that meets the target will be assigned one point. If all outcomes meet the targets, a maximum score of 3 points will be assigned to the patient composite score and a maximum score of 9 points will be assigned to the process composite score. If we cannot find any data about the process variable, we will assume that it did not happen. A variable that improves during the intervention period will be scored as +1; each variable that worsens will be scored as −1; and each variable that does not change will be scored as 0. As such, composite scores can be grouped into three categories: worsened, unchanged or improved.

The follow-up period will be 12 months. Outcomes will be collected at five points: before the start of the study, at 3 months, at 6 months, at 9 months and at 12 months, with the differences between pre- and post-implementation forming the outcome scores. A mean will be calculated for the intervention and control groups. The trial will be terminated at one of the interim analysis points when a mean difference of 0.3% might be reached for the primary outcome of HbA1c change.

### Process evaluation

In the process evaluation, actual use of the system as well as users’ perceptions about the feasibility of the electronic decision support will be measured. These aspects are two necessary components of a process evaluation [22].

Scripts are automatically triggered, and users can see that a script is available from their main screen but have to click to view the script content. As such, click events can give us an indication of physicians’ interest in using the system. A new record is inserted in the log file for each script that is triggered. The following data of actual use are collected in a log file at the physician level: identification code of the script; type of script that is triggered and opened (Reminder, Interaction, Contra-indication, Drugs to avoid, Indication, GuideLine Link); script node expanded (yes/no): *e*.*g*., a long reminder displays when a short reminder is clicked; request for the script information (yes/no).

As such, the percentage and the type of scripts that were opened will be calculated, as well as the percentage of scripts that were expanded and the percentage of scripts for which the script information was requested.

Process information regarding actual use of the Evidence Linker will be logged in a similar way.

### Physicians’ perceptions of the reminders

Physicians get the opportunity to give their opinion when a reminder is triggered. Physicians can choose between useful, not useful, or no advice. They can give feedback for each reminder and can indicate if they wish to block the reminder in the future.

### Data collection

Data will be automatically extracted from the EMR of the family physician for all patients who satisfy the eligibility criteria. Data will be extracted at five points in time: before the start of the study, at 3 months, at 6 months, at 9 months, and at 12 months. All outcomes can be reliably determined from the electronic medical records. When a physician closes HealthOne, a reminder will be shown to export the data. The family physician can visualize the data and has to confirm if he agrees to export. Modifications to this file cannot be made by the physician.

Identification data of the patient will be manually converted to an output code via a hashing program installed on the computer of the family physician. Data will be sent by mail to an independent third person. This person will be responsible for uncoupling the identifiable data of the family physician (name and e-mail address) from the patient data and the data related to the actual use of the system. A meaningless code will be allocated to the physician’s data and its corresponding patient and use data. The coupling between these data will be exclusively possessed by this person and will be used to couple the data at the different points in time.

These coded data will be sent securely to the researcher. Patient data will be sent to the researcher at the five interim analysis points. Data concerning EBMeDS use will be sent once to the researcher at the end of the study period after patient data are analyzed. Data related to system use will be used to explore possible relationships between the patient outcomes and the actual use of the system. As there is only one file of actual use for the physicians in the intervention group, the researcher would not be blinded if these data were sent together with the patient data.

### Sample size

Based on the data in the literature, a mean difference of 0.3% in HbA1c change could be considered clinically significant (SD of HbA1c change 1.3%) [23-25]. To detect a mean difference of 0.3% in HbA1c change at four post-treatment occasions, with a two-sided unpaired *t*-test, a 5% family-wise significance level and Bonferroni correction for multiple testing, 1:1 allocation and a power of 80%, a sample size of 463 patients in each group will be necessary, assuming a withdrawal rate of 10%. Additional adjustment for clustering of patients within physician practices by the design effect [26], assuming 10 patients per cluster and an intra-class correlation coefficient of 0.047 based on data in the literature [27], leads to a sample size of 659 patients in each arm. Sample size calculations were based on historical data. They can be adjusted after baseline registrations if correct standard deviations and intra-cluster correlation coefficients are known. Sample size calculation was performed using SAS software.

### Recruitment

All Dutch- and French-speaking family physicians who use HealthOne in their daily practice will be informed about the study and will be sent an e-mail invitation to participate by one of the researchers. Physicians who do not react to the e-mail after 14 days will be sent a reminder. Any physicians who do not react to the second reminder will be telephoned by one of the researchers after another seven days.

### Randomization

Physician practices that agree to participate will be randomly assigned to one of the two groups following simple randomization procedures. Randomization will be performed by a statistical consultant with an electronic random list generator, anonymously for the researchers. Allocation will be concealed from the participants until the study start and after physicians’ informed consent is obtained. Physicians will be aware of the allocated arm; patients will not be informed about the intervention. Data analysts will be kept blinded to the allocation. Outcomes are objective measures directly extracted from the electronic medical records.

### Data analysis

Descriptive statistics will be provided regarding baseline variables of the patients and the family physicians. Intervention and control groups will be compared regarding baseline variables to evaluate the randomization. Linear mixed models will be used for continuous patient-level variables, with a random intercept to account for clustering of patients within physician practices. Logistic regression models based on generalized estimating equations (GEE) to account for clustering will be used for binary patient-level variables.

Differences between intervention and control groups regarding continuous outcome measures (change versus baseline in HbA1c, cholesterol levels, and blood pressure) will be assessed using linear mixed models (multilevel regression models) with random intercepts for physician practices and random intercepts for patients to account for repeated measurements over time. The primary fixed effect of interest will be the difference between intervention and control group, which will be tested at each time point (3 m, 6 m, 9 m and 12 m). If important differences in baseline characteristics are demonstrated between both groups, we will account for that by including the relevant variables in the model.

Differences between intervention and control groups regarding three-level composite scores (worsening, unchanged, improvement) will be assessed using random-effects proportional odds models, or, in case the proportional odds assumptions do not hold, baseline-category logits models, with random intercepts for physician practices and random intercepts for patients to account for repeated measurements over time. As with the analysis of continuous outcomes, differences between intervention and control groups will be tested at all time-points, and correction for imbalance in baseline characteristics will be applied if indicated.

To study the effect of the actual use of the EBMeDS system on the outcome measures, models similar to those described above will be used for analyzing the effect of intervention. However, they will now include a measure for the use of EBMeDS as the main explanatory variable, and will restrict analysis to the data obtained in the intervention group.

### Ethics

Before the start of study, we will present the physicians with an informed consent that outlines the EBMeDS intervention and the purpose of the trial to study the effectiveness of the system.

Individual informed consent from the patient cannot be sought. Reminders show up unexpectedly during practice when patient data deviate from evidence-based targets. It is not practical to ask for informed consent at these moments. Furthermore, reminding the physician of the informed consent forms at every consultation may bias the study results in favor of the intervention.

Data exports will be compliant with privacy legislation. Registration of the data processing to notify the Belgian Privacy Commission was performed on the commission website: http://www.privacycommission.be. Authorization for the use of medical and personal data was requested from the Sector Committee of Health.

Ethics committee approval for this study was obtained from the University Hospitals Leuven Medical Ethics Committee in May 2013.

## Discussion

The strength of the EBMeDS system is its platform-independent, service-oriented architecture with strong central management. This technical and organizational structure makes it possible to manage the evidence and the system itself on a national scale. Furthermore, this structure facilitates gradual integration in other Belgian software packages and other healthcare settings.

The first steps towards an open environment of continuous system improvement are taken with this study. End-users’ perceptions of the system will provide further understanding of their information needs to improve the feasibility of the system. The results of this study will be applicable at a national level where system adaptations should be made.

Although the EBMeDS system covers all clinical areas of disease, analysis will be limited to diabetes outcomes. We chose to start with the analysis of diabetes outcomes as clear recommendations on the basis of high levels of evidence exist for this pathology, but there is still a large gap between recommended care and the care that patients actually receive. We opt to display relevant reminders in all clinical areas, not just for diabetes. Our aim in so doing is to avoid drawing the attention of the physician to diabetes and to attain full operationalization of the system in a real-life situation in which all clinical areas are covered.

We are aware that the success of the system will depend on the quality of the data-coding and stable use of the EMR. Before the start of the study, we planned to correctly reorganize and code the medical records to guarantee a clear picture from the start. We finally decided not to proceed with the reorganization of the records as this might not reflect a real-life situation. However, we will investigate this as a possible shortcoming of the implementation process and will use this information in the evaluation of the feasibility of the system and as a possible component of future training initiatives.

## Competing interests

AH, SVDV, and RVDS are salaried editors of EBMPracticeNet, the organization responsible for the implementation of EBMeDS in Belgian family medicine.

## Authors’ contributions

All authors were involved in determining the research question, the study design and the methodology. AH wrote the protocol, BA supervised the process. All authors commented on subsequent drafts of the protocol and approved the final version.
